# Exceptionally Strong Double-Layer Barriers Generated by Polyampholyte Salt

**DOI:** 10.1021/acs.jpcb.5c00012

**Published:** 2025-04-03

**Authors:** David Ribar, Clifford E. Woodward, Jan Forsman

**Affiliations:** † Computational Chemistry, Lund University, P.O.Box 124, S, Lund 221 00, Sweden; ‡ School of Physical, Environmental and Mathematical Sciences, ADFA Canberra ACT, University of New South Wales, University College, Canberra 2600, Australia

## Abstract

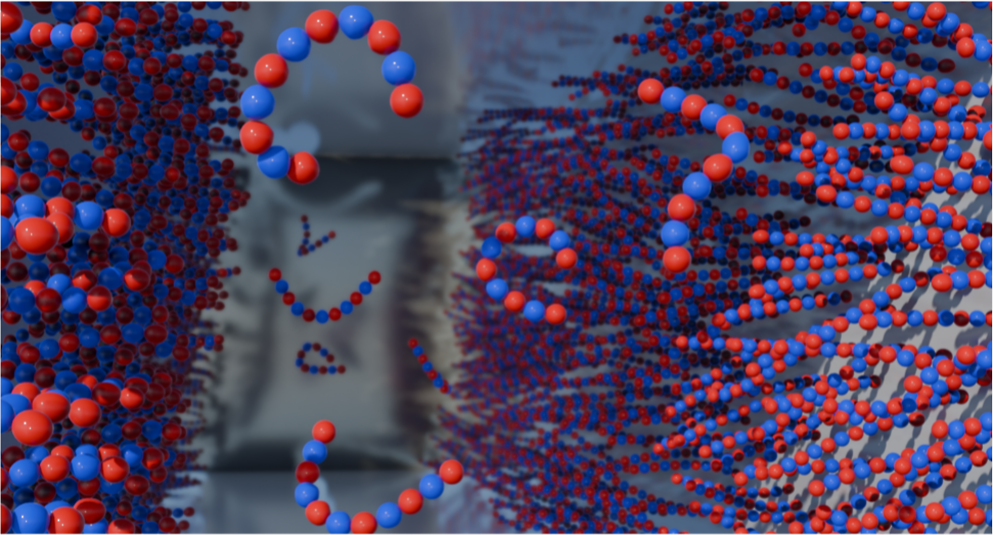

Experiments using the surface force apparatus have found anomalously long-range interactions between charged surfaces in concentrated salt solutions. Ion clustering has been suggested as a possible origin of this behavior. In this work, we demonstrate that if such stable clusters indeed form, they are able to induce remarkably strong free energy barriers under conditions where a corresponding solution of simple salt provides negligible forces. Our cluster model is based on connected ions producing a polyampholyte salt containing a symmetric mixture of monovalent cationic and anionic polyampholytes. Ion distributions and surface interactions are evaluated utilizing statistical–mechanical (*classical*) polymer density functional theory, cDFT. In the Supporting Information, we briefly investigate a range of different polymer architectures (connectivities), but in the main part of the work, a polyampholyte ion is modeled as a linear chain with alternating charges, in which the ends carry an identical charge (hence, a monovalent net charge). These salts are able to generate repulsions, between similarly charged surfaces, of a remarkable strength, exceeding those from simple salts by orders of magnitude. The underlying mechanism for this is the formation of brush-like layers at the surfaces, i.e., the repulsion is strongly related to excluded volume effects, in a manner similar to the interaction between surfaces carrying grafted polymers. We believe our results are relevant not only to possible mechanisms underlying anomalously long-ranged underscreening in concentrated simple salt solutions but also for the potential use of synthesized polyampholyte salt as extremely efficient stabilizers of colloidal dispersions.

## Introduction

Using simple salt to regulate the stability of colloidal dispersions that contain charged particles is a well-established process, with a firm theoretical foundation.^1−5^ Mean-field treatments of aqueous systems tend to be accurate at low and intermediate concentrations when the salts are composed of monovalent species. An important mean-field result is the so-called Debye screening length, λ_*D*_, that describes the effective range of electrostatic interactions in the presence of salt. As the salt concentration increases, *ionic screening* leads to a reduced range, with λ_*D*_ ∼ 1/*c*^1/2^. However, recent experiments using the surface force apparatus, SFA, indicate a peculiar nonmonotonic behavior: above a threshold concentration (usually around 1M), the range of electrostatic forces *increases* upon the addition of salt.^6−9^ This remarkable response has some support from colloidal stability^10^ and thin film^11^ investigations, but there are also contradictory experiments.^12^ Several theoretical efforts have been made to establish possible underlying molecular mechanisms,^13−23^ but there is still no broadly accepted view on the matter.

Ion pairing, or ion clusters, have been suggested to play a role for the alleged strong and long-ranged repulsion at high ionic strengths.^6,21−26^ There are also some experimental results that more directly suggest the formation of ion clusters at high ionic strength.^27,28^ In this work, we investigate surface forces in the presence of aqueous solutions that contain *constructed* “ion clusters”, or at least molecular architectures that can be viewed as simple models of monovalent ion clusters. Our description is based on an implicit treatment of water, which enters only via its dielectric constant, ϵ_*r*_ = 78.3, and ions that are connected to form “ion clusters” with a univalent net charge. This system will be treated using *classical* polymer density functional theory, cDFT. As we demonstrate below, these systems can generate exceptionally strong repulsive interactions between similarly charged surfaces at short and intermediate separations.

While the polyampholyte chains that we investigate are assumed to be linear, we demonstrate, in the Supporting Information, that very similar results are obtained with a branched (star-like) architecture.

## Model and Theory

Our model polyampholytes are composed of connected charged hard spheres with a diameter *d*. The bond between neighboring charges in a cluster has a fixed length *b* and allows for full rotationally flexibility. The solution is modeled using an implicit solvent and a salt composed of an equimolar mixture of monovalent polyampholyte cations and anions. The number of charged monomers per chain is denoted by *r*, with each *r*-mer carrying a net single elementary charge. In the main article, we consider a simple linear architecture with alternating charges along the chain. Thus, the polyampholytic cation will have positive end monomers, and a total of (*r* + 1)/2 positive and (*r* – 1)/2 negative charges, with the opposite true for anions. Note that *r* must be an odd number, otherwise the polymers become net neutral. Here, we compare surface interactions in the presence of a purely polyampholyte salt with those obtained with a simple salt.

**Figure 1:**
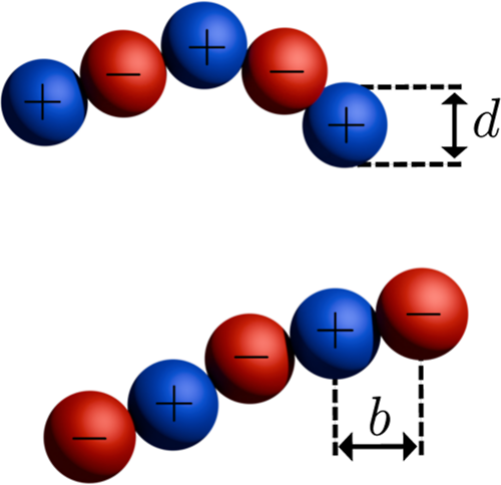
A graphical illustration of a cation and an anion in a 5-mer polyampholyte salt.

In Figure 1, we present a cartoon illustration of our polyampholyte salt model.

This is for a case where the hard-sphere diameter *d* equals the bond length *b*, but we also consider cases with smaller monomers.

In the Supporting Information, we explore different chain architectures and charge distributions. For example, we investigated the situation where the charges are collected in blocks of monomers of the same valency. Such chains likely adopt a folded state unless there is additional simple salt. These architectures appear to give rise to rather dramatic effects, but we postpone their more detailed study for future work.

Nonbonded monomer–monomer interactions are denoted by ϕ_*ij*_(*r*), where
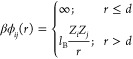
1Here,  is the inverse thermal energy, *l*_B_ ≈ 7.16 Å is the Bjerrum length at 298 K, and *Z*_*i*_ and *Z*_*j*_ denote the valencies of interacting monomers *i* and *j* (|*Z*| = 1 in all cases), respectively. Monomer densities are denoted as *n*_+_ (cations) and *n*_–_ (anions).

We study the interactions between two flat, hard, and infinitely large surfaces, carrying a surface charge density σ = – 1/70*e*/Å^2^, where *e* is the elementary charge. This is a value typical to mica,^29^ i.e., the surfaces used in SFA experiments. The surfaces are immersed in our model mixture, and the separation *h* between the surfaces is varied. The system is treated using the grand canonical ensemble, i.e., the confined fluid is in chemical equilibrium with an infinite bulk. The equilibrium (minimal) free energy is obtained at each separation, and the net osmotic pressure is evaluated either as a discrete free energy derivative or from the monomer contact values at the surfaces. All polymer configurations are accounted for,^30^ subject to a Boltzmann weight, under the assumption that densities only vary in the direction transverse to the surfaces.

For a completely ideal bulk polymer solution, in which there are no particle–particle interactions or external fields, save the bond constraints, the polymer free energy, , for an *r* – *mer* can be *exactly* written as
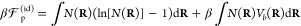
2where a polymer configuration is represented by **R** = (**r**_1_, ..., **r**_*r*_) and the density distribution *N*(**R**) is defined such that *N*(**R**)d**R** is the number of polymer molecules having configurations between **R** and **R** + d**R**. *V*_*b*_(**R**) is the bond potential between the connected monomers. In this work, we only consider bonds of fixed length, i.e., , where *b* is the bond length and δ(*x*) is the Dirac delta function.

For our model of a bulk polyampholyte salt, the Helmholtz free energy is given by
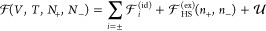
3We have used the index *i* to indicate that there are two types of (nonideal) chains with alternating charges, one of which (the cation) starts and ends with a positive charge. Anions will have negative ends. The *ideal* contribution to the free energy in the bulk is identical for both species, but in a heterogeneous environment, they will be different. Excluded volume interactions are approximated by  and are assumed to depend only on total cationic and anionic monomer density *n*_+_, *n*_–_. We have used the generalized Flory dimer (GFD) theory to estimate this term.^31,32^ The GFD theory takes into account that end monomers exclude less volume than dissociated monomers and that central monomers exclude even less volume. All electrostatic interactions are collected in  and treated at the mean-field level. In our slit environment, we use the grand potential Ω, which can be expressed using the following Legendre transformation
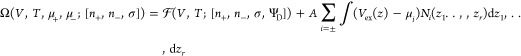
4Here, *z* is the direction normal to the surfaces, *A* is the surface area, and Ψ_D_ is a Donnan potential that ensures that the system is electroneutral. Polymer configurational entropy, excluded volume, and electrostatic mean-field interactions (including a wall–wall repulsion) are all contained in . The cation–anion chemical potential is denoted by μ_+_/μ_–_. *V*_ex_ represents the nonelectrostatic interaction with the confining surfaces, and as mentioned, these are purely steric in nature, so *V*_ex_(*z*) = 0 if d/2 < *z* < (*h* – d/2) and infinite elsewhere. The grand potential was minimized numerically at each separation *h* to its equilibrium value, Ω_eq_(*h*), using Picard iterations. Defining *g*_s_(*h*) ≡ Ω_eq_/*A* – *p*_b_*h*, where *p*_b_ is the bulk pressure, we arrive at the *net* interaction free energy as Δ*g*_s_ ≡ *g*_s_(*h*) – *g*_s_(*h* → ∞).

Details on the numerical procedure can be found elsewhere,^33,34^ but we provide a brief description. First, we note that the planar symmetry, combined with our mean-field assumption, allows us to integrate densities and interactions along the (*x*, *y*) directions parallel to the surfaces, taking proper account of the electroneutrality criterion. We simplify the notations by simply stating that only *z* positions within the slit are relevant, i.e., in the vicinity of a surface, integrals are truncated by the surface. The (*x*, *y*)-integrated Coulomb interaction energy, ϕ_*C*_(*z*, *z*′), between charges with valency *Z*_γ_ and *Z*_δ_, located at positions *z* and *z*′, is βϕ(*z*, *z*) = −2π*Z*_γ_*Z*_δ_*l*_B_|*z* – *z*′|. Let us define , where we note that cationic and anionic charges have equal size. All polymer configurations are accounted for by cDFT, subject to a mean-field Boltzmann weight. The equilibrium monomer density can be expressed in terms of auxiliary fields *c*(*i*, *z*), with *i* denoting the monomer position along the chain. In our case, we need two separate fields of this kind, *c*_+_(*i*, *z*) and *c*_–_(*i*, *z*), with the index denoting the net polymer charge. Let us illustrate the procedure for cationic chains. The contribution from cationic polymers to the *total* monomer density *n*′(*z*) at *z* can be written as
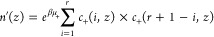
5where the prime index highlights that this is only the contribution from net positive chains. The auxiliary functions are related by a recursion relation
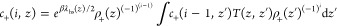
6for *i* > 1 and
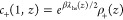
7Here, ρ_+_(*z*) ≡ *e*^–βeΨ(*z*)/2^, where Ψ(*z*) is the local potential (including the Donnan potential) at *z* and *e* is the elementary charge. *T*(*z*, *z*′) manages the fixed bond length constraint between connected monomers, including a normalization constant
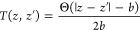
8

Net negative polymers are treated in an analogous fashion. Conservative Picard iterations are utilized, where a small fraction of newly predicted densities are mixed with “old” ones. A similar procedure applies for the Donnan potential, which also needs to be incrementally adjusted so that the predicted density distributions are commensurate with the criterion of global electroneutrality. Our convergence criterion is that the maximum relative difference between “new” and “old” predictions is less than 10^–7^.

## Results

In order to compare with published SFA data, we will utilize the Derjaguin approximation and transform calculated net free energies per unit area to the force per radius, *F*/*R*, appropriate to the crossed cylinder setup in experiments. In that case, *F*/*R* = 2πΔ*g*_s_. The net pressure acting perpendicular to the flat surfaces is denoted *p*_net_, with *p*_net_ = – ∂Δ*g*_s_/∂h.

**Figure 2:**
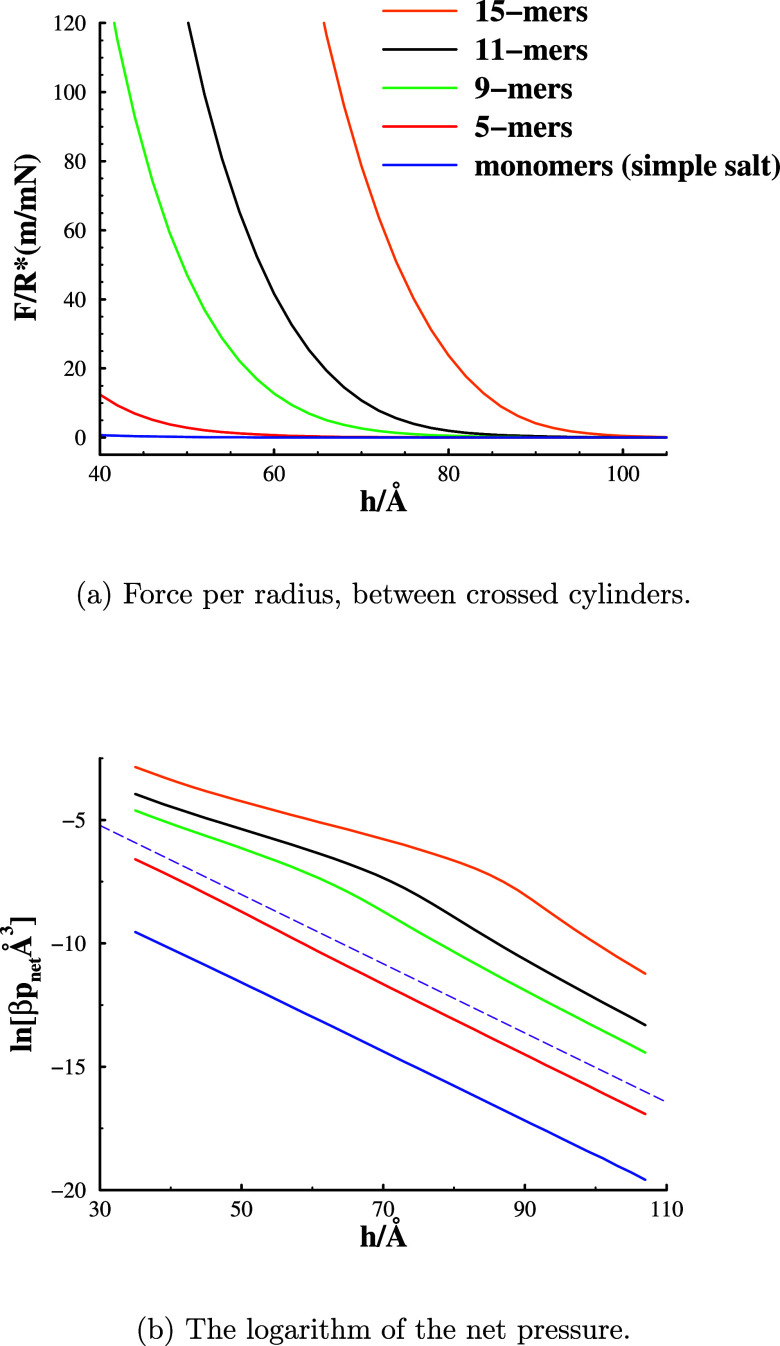
Surface interactions at 182 mM salt concentration for simple salts and polyampholytes of various lengths. Note that the concentration of charged monomers increases with the polymer length. For instance, with 11-mers, the bulk concentration of cationic as well as anionic monomers is 2M, but the polyampholyte *salt* concentration is still 182 mM. Legend colors are the same in graph (b) as in (a). The thin dashed line in graph (b) shows a line with slope −1/λ_D_(182 mM), where λ_D_(182 mM) is the Debye estimate of the screening length (about 7.1 Å), for a 182 mM aqueous solution with a monovalent salt. The placement along the *y*-direction of this dashed line is arbitrarily chosen.

In Figure 2, we compare surface interactions for simple salt and polyampholyte salt at a bulk salt concentration of 182 mM. In the latter case, we consider monodisperse chains of increasing degree of polymerization, while the chain concentration remains fixed at 182 mM in the bulk. Such a scenario could serve as a crude approximation to a hypothetical system where ionic clusters begin to form in a simple monomeric salt (at 182 mM) and any added salt only serves to increase cluster size. This type of behavior was actually observed in recent all-atomistic MD simulations by Komori and Terao.^25^ They found that increasing the concentration of monovalent simple salts beyond a critical value (in that case, 1M) gave only an insignificant increase in the free ion concentration.

In Figure 2a, we see a significant increase of the repulsive interaction between the charged surfaces compared to monomeric salt, even for rather modest cluster sizes. While the polyampholyte chains are assumed to be linear, in the Supporting Information, we report that quite similar results are obtained with a branched (star-like) architecture. Despite the growth in repulsion with chain length, the long-range decay of the interaction remains relatively constant. In Figure 2b, we see that the long-range decay is very similar in all cases. For the case of the charged monomers (simple salt), the asymptotic decay appears to be exponential, with a decay length that agrees exactly with the Debye length. This is to be expected as the *c*DFT uses mean-field electrostatics. On the other hand, there is a slight but noticeable decrease in the asymptotic decay length for the polyampholyte systems, with the decay length decreasing with cluster size. This is due to the cooperative adsorption of chains at the charged surface, which leads to more efficient screening of the surface charge than that for monomeric salt ions. We also notice that at the intermediate range, polyampholyte-mediated repulsion decays more slowly than with simple salt. The reason for this is discussed below.

**Figure 3:**
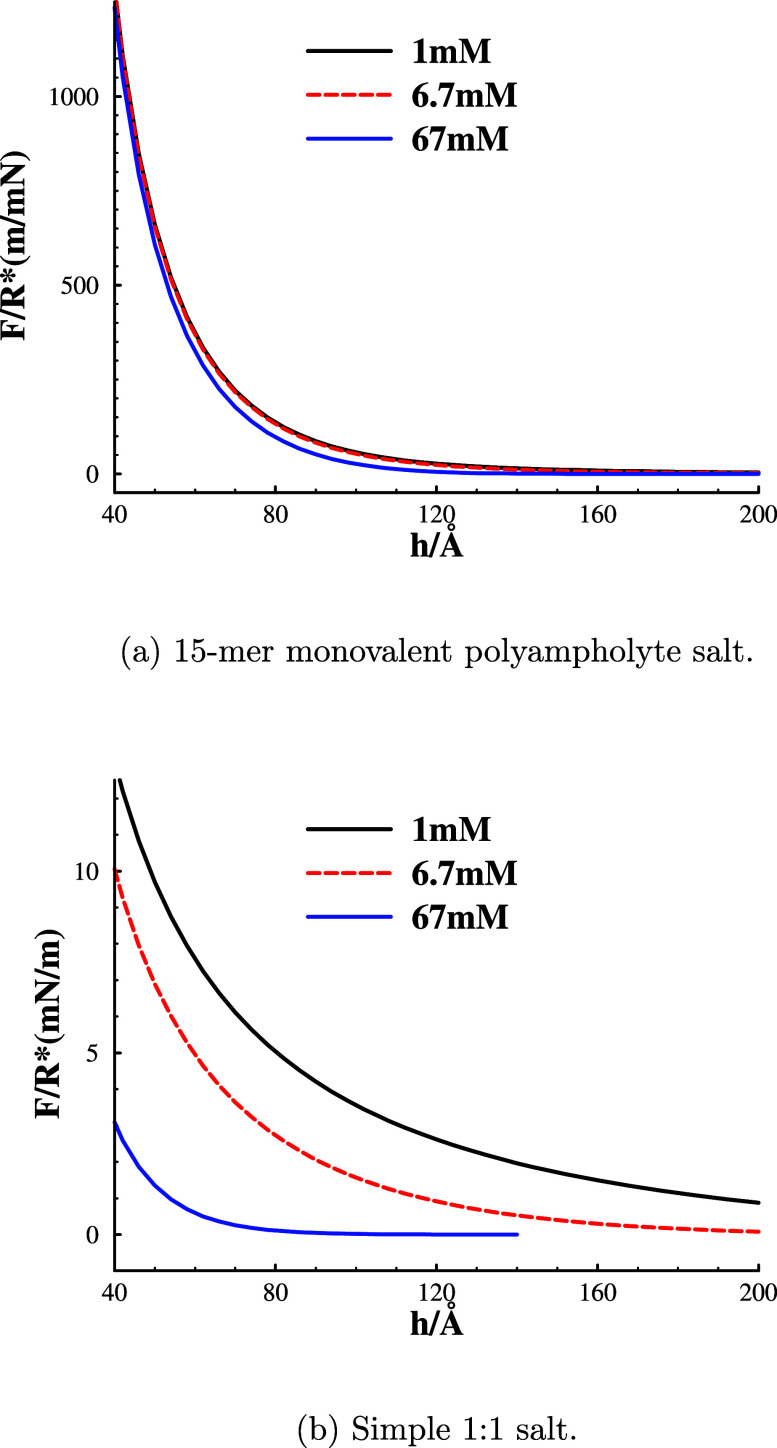
Concentration dependence. Interaction free energies for polyampholyte (graph(a)) and simple (graph(b)) salts. Note the difference in scale between graphs (a) and (b).

Another interesting property of polyampholyte salt systems is a remarkable insensitivity to changes in the concentration at short and intermediate separations. This is shown in graph Figure 3a, where we see how a 67-fold increase of the polyampholyte concentration leaves the surface interactions almost unchanged. This is in stark contrast to the response of simple salt solutions, illustrated in Figure 3b, where the same concentration increase almost eliminates the repulsion. Note the 2 orders of magnitude difference in scale for the displayed interaction curves.

**Figure 4:**
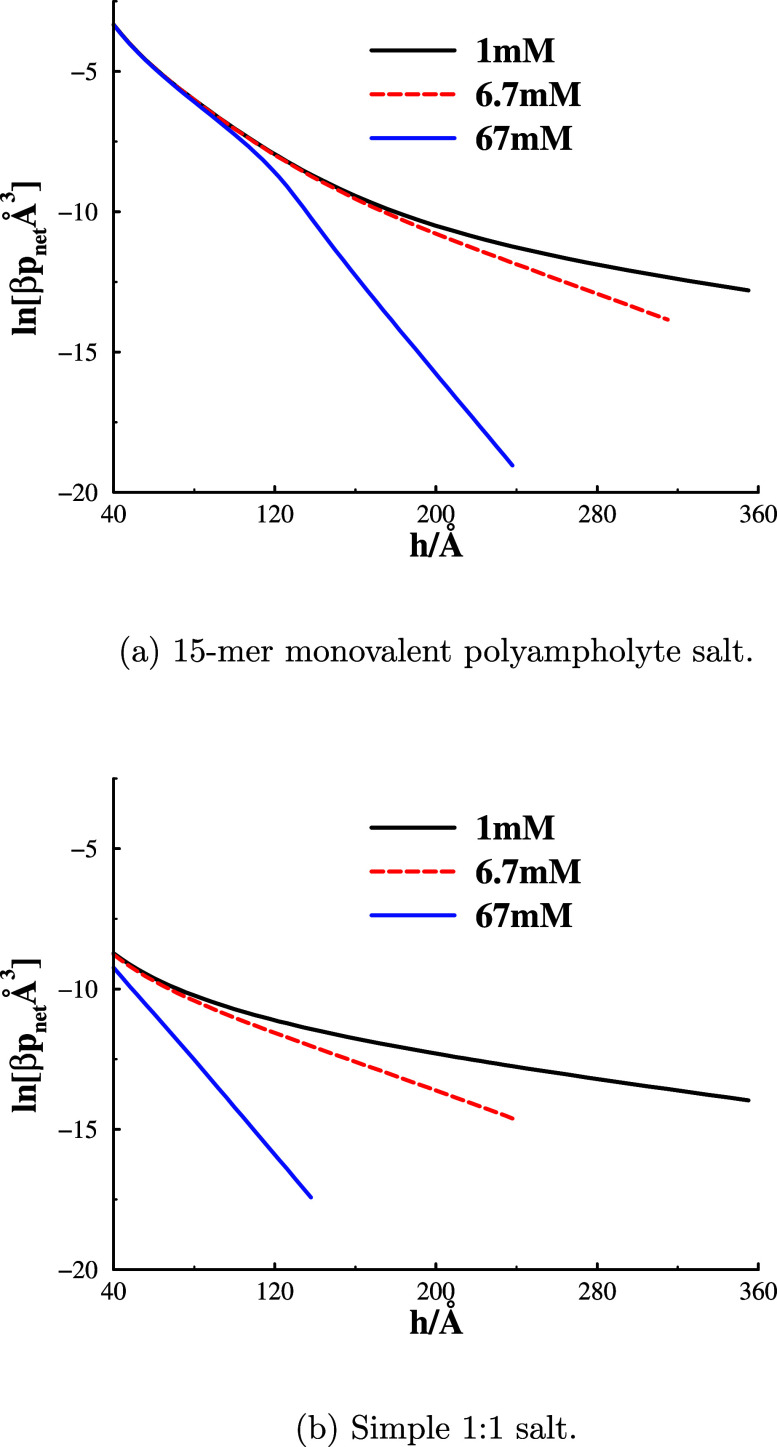
Concentration dependence of ln[net pressure] for polyampholyte (graph(a)) and simple (graph(b)) salts.

This does not mean that the long-range decay length at large separations is insensitive to the concentration of polyampholyte salt though. In Figure 4a, we see that an increase in the polyampholyte salt concentration leads to a considerably faster decay at *large* separations. As noted above, comparisons show that the *long-ranged* decay length is very similar for the polyampholyte and simple salts, Figure 4b.

**Figure 5:**
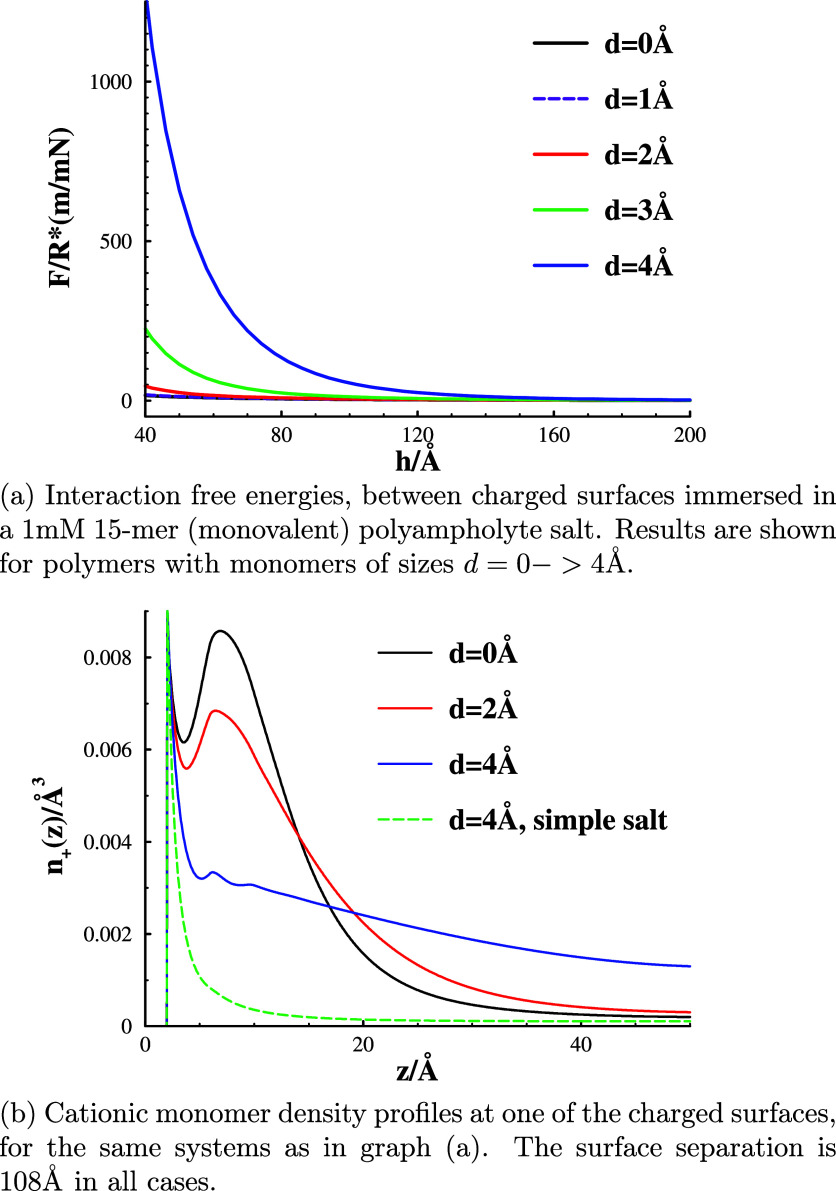
Excluded volume effects, evaluated by changes to the monomer hard-sphere diameter. The bulk polyampholyte salt concentration is 1 mM.

So, where does the very strong repulsion at intermediate separations seen in the polyampholyte salts originate from? It turns out that steric interactions play a crucial role. This is exemplified in Figure 5a, where we note that a reduction of the monomer hard-sphere diameter leads to a dramatic drop of the surface force. This is, in turn, related to the monomer density distribution at the surfaces, as shown in Figure 5b. Only cationic monomer densities are displayed, but the overall trend is the same for anions (separate distributions are provided in the Appendix, for the case *d* = 4 Å). As the monomer radii decrease, the chains adopt a much more compact density profile. This suggests weaker chain–chain overlap as the surfaces are brought closer together. Moreover, the smaller size of the monomer leads to a weaker excluded volume free energy penalty for a given degree of chain–chain overlap. This chain overlap mechanism is, to some extent, similar to that resulting in strong repulsions between surfaces covered by polymer brush layers. As mentioned, we make direct comparisons in the Appendix.

However, there is another mechanism that generates a strong and long-range repulsion in the polyampholyte systems. This mechanism is also related to excluded volume but in a more indirect sense. The presence of adsorbed layers of net cationic (monovalent overall) polyampholytes leads to a strong exclusion of anionic polymers, i.e., an extremely low salt concentration, in the slit regime. As a consequence, the polyampholyte system effectively behaves as a “counterion only” case (except at very large separations), which is known to display double-layer interactions with a very slow decay. One way to illustrate this is to compute the “apparent” surface charge density, σ_app_, defined as
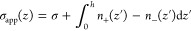
9

**Figure 6:**
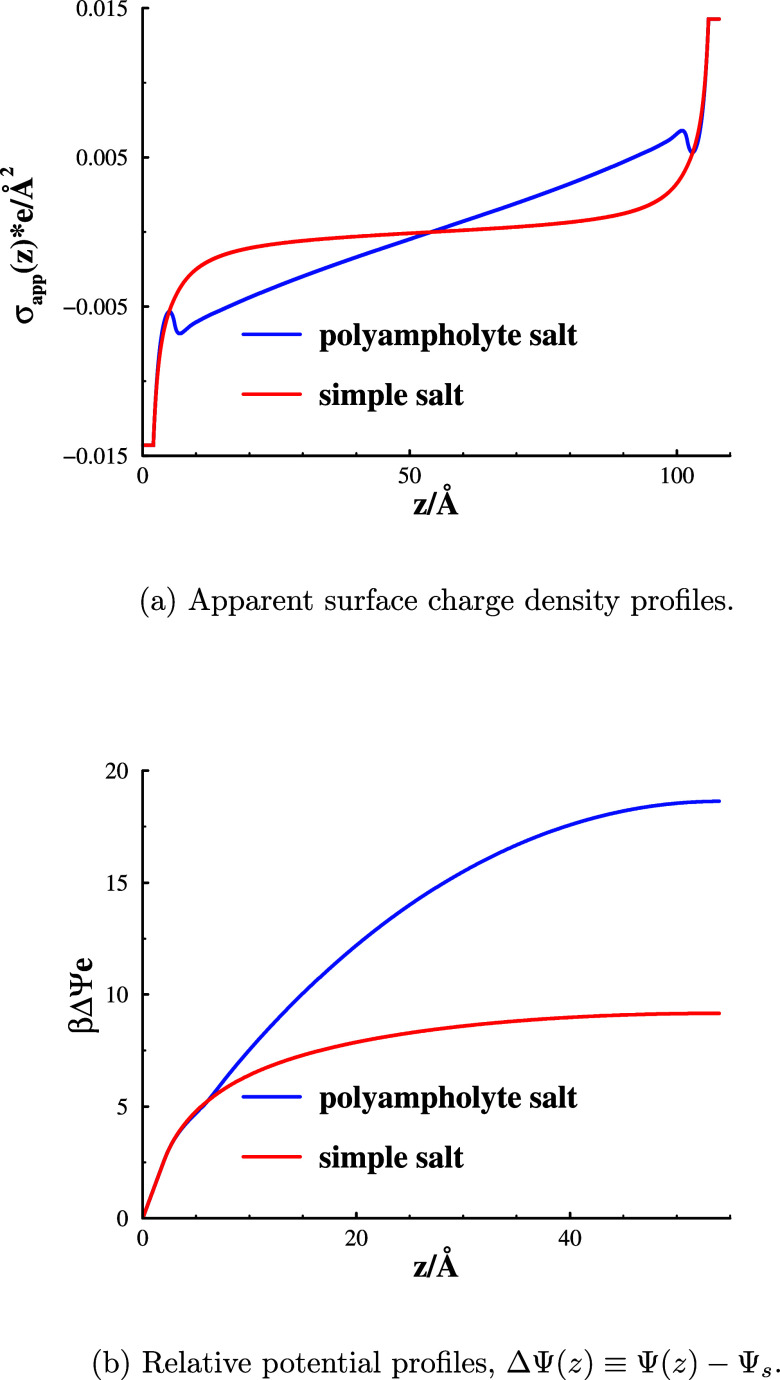
Apparent surface charge density (graph (a)) and relative potential (graph (b)) profiles for 1 mM salt solutions at a separation of 108 Å. The blue curve shows data for 1 mM 15-mer polyampholyte salt, whereas the red curve shows corresponding results for a simple 1:1 solution at 1 mM.

Such profiles are compared in graph (a) in Figure 6. The drop of σ_app_(*z*) away from the surface is rather steep, even at 1 mM, in the presence of the simple salt. But in the polyampholyte solution, it is linear throughout the slit, except very close to the surfaces. This results from an almost complete lack of polyampholyte *salt* (co-ions to the surface) in the intersurface regime. We can quantify this by calculating the ratio between the integrated polymer cation density and the integrated polymer anion density (note: polymer ratios are not monomer ratios). This ratio has an enormous value of about 5.6 × 10^12^ at a separation of 108 Å. The corresponding ratio for the simple salt is only about 3.0 × 10^4^. Another consequence of the near complete salt exclusion with polyampholytes is a larger potential difference between the mid plane region of the slit and the surface. Denoting the local potential by Ψ(*z*) and the surface value by Ψ_s_, we have compared potential difference profiles in graph (b) of Figure 6. Outside the charge-excluded regime near the plane of charge (where there is a trivial linear variation), the potential difference saturates much faster in the presence of a simple salt, in which case there is a significant amount of co-ions (anions) in the slit. In summary, since the polyampholyte cations exclude a large volume, they form a wide brush-like layer at each surface, and co-ion polymers are effectively excluded from the slit by a combination of electrostatic and excluded volume interactions. In a similar fashion, the counterion layers themselves repel each other strongly and with a weak decay due to steric as well as electrostatic forces, where the latter behave similarly to those of counterion-only double layers (except at very large separations).

## Conclusions

The mechanism underlying the very strong repulsion that we observe in these systems is similar to that between surfaces with adsorbed polymer layers. In that case, we know that the range of the surface interactions is determined by the chain length. So why is this interesting? We have seen (Figure 2b) that chain–chain interactions can give rise to an intermediate decay length that is longer than the Debye length. Though our results show that the Debye length (albeit slightly modified) was relevant at very large surface separations, our results may still have relevance to the anomalous interactions found in SFA measurements in simple concentrated salts. In this work, we have limited ourselves to monodisperse chains of finite length. Thus, at very large surface separations, the overlaps between finite chains are less important than electrostatic effects, and this is why the usual Debye-like decay of the interactions eventually prevail asymptotically. For aggregating clusters, the chain size distribution would likely have an exponential form, and in this case, chain overlaps remain significant at much larger surface separations, possibly dominating the electrostatic mechanism asymptotically and giving rise to apparently anomalous long-range decays.

We envisage that our theoretical predictions are readily verifiable by surface force measurements, for instance, using polypeptides that are composed of alternating amino acids of opposite charge (at some suitable pH). Upon verification, such polyampholyte salt systems could be further explored in the context of colloidal stability.

## Figures and Tables

**Figure 7:**
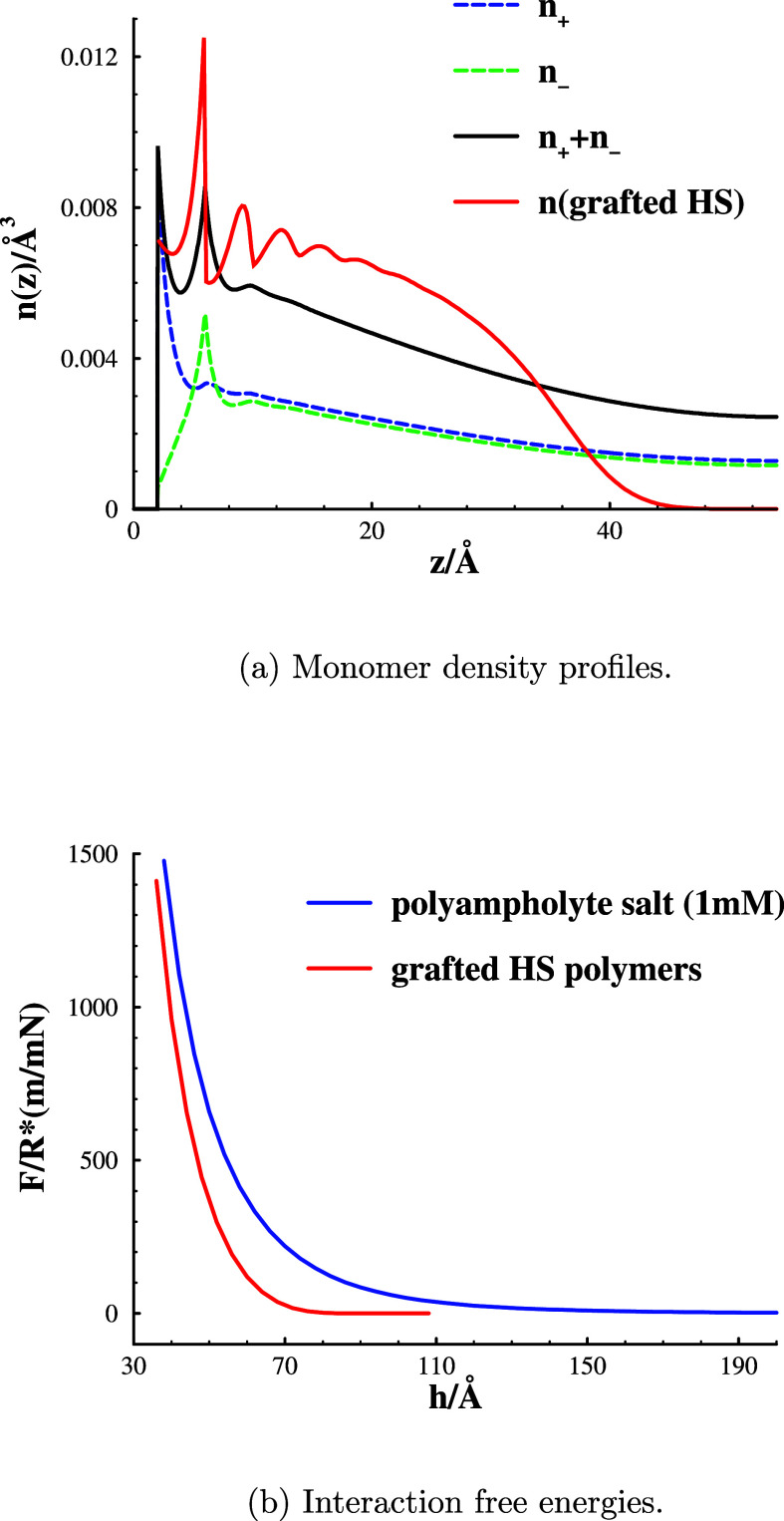
Comparisons between polyampholyte-mediated interactions between charged surfaces (15-mers, with *d* = *b* = 4 Å), and interactions between neutral and inert surfaces carrying grafted chains, which in turn are composed of hard-sphere monomers (also with 15-mers, with *d* = *b* = 4 Å). The grafting density in the latter case is adjusted to a matching integral of the total monomer density, at this separation (*h* = 108 Å).

## Data Availability

Github repository: the code used for cDFT calculations pertaining to the main article, along with the data generated, is freely available at: https://github.com/janneforsman/Polyampholyte_pure/releases/tag/v1.0.

## Appendix

### Comparisons with Surfaces Carrying End-Grafted Neutral Polymers

Here, we compare the polyampholyte-mediated forces between charged surfaces that we have focused on in this work to interactions between neutral surfaces covered by end-grafted chains of connected hard-sphere monomers. In the latter system, we anticipate a repulsion at short-range, which is almost entirely steric in origin (save a small ideal contribution). This makes it suitable as a “steric reference”. Our polyampholyte system is of course different, but it is nevertheless instructive to compare with a simpler system, having “purified” steric interactions.

Consider the *d* = 4 Å system in Figure 5 (1 mM polyampholyte salt). Graph (b) of this Figure shows cationic monomer density profiles at a separation of 108 Å. In order to arrive at a grafted surface “correspondence”, we have used the integral of the total monomer density, *n*_+_ + *n*_–_, as a guide and adjusted the end monomer grafting density so that the integral of the hard-sphere monomer density has the same value. As in Figure 5, we consider 15-mers, with *b* = *d* = 4 Å. The grafted chains have one monomer constrained to a region within one bond length (*b*) from the surface.

The resulting density profile of hard-sphere monomers (red curve) can be compared to the density profile of the total polyampholyte monomer density (*n*_+_ + *n*_–_, black curve) in Figure 7a. We note that the hard-sphere monomer density is roughly constant with some oscillations, up to a distance of about 20 Å from the surface. Beyond this distance, the hard-sphere monomer concentration drops rapidly. The corresponding decay is significantly slower for the polyampholyte monomers, which in turn is related to the polyampholyte salt exclusion discussed in relation to Figure 6. Nevertheless, there is certainly some resemblance between the curves. Corresponding interaction free energies are displayed in Figure 7. As expected from the density profiles, the polyampholytes mediate a more long-range repulsion, but given that the two model systems are completely different, one could argue that the difference is relatively small. This lends some further support to the suggestion that the remarkably strong interactions that we have observed between charged surfaces, in the presence of polyampholyte salt, have a strong steric contribution, akin to that from polymer brush layers. It should, however, be emphasized that the steric effects are subtler for the salt solution since they also affect the electrostatic interactions, as discussed in the main paper.

## Supporting Information Available

Results obtained with other modeling options for monovalent polyampholyte salts (PDF)
