# Supplementary material for: Exceptionally Strong Double-Layer Barriers Generated by Polyampholyte Salt
Source: J Phys Chem B. 2025 Apr 3;129(17):4241–8. doi: 10.1021/acs.jpcb.5c00012 (PMC12051195; doi:10.1021/acs.jpcb.5c00012)
Supplement: Supplementary file 1 — jp5c00012_si_001.pdf [file jp5c00012_si_001.pdf]

Supporting information for:

Exceptionally strong double-layer barriers generated by polyampholyte salt

David Ribar[a], Clifford E. Woodward[b], Jan Forsman[a,*]

[a] *Computational Chemistry, Lund University, P.O.Box 124, S-221 00 Lund, Sweden*

[b] *School of Physical, Environmental and Mathematical Sciences University College, University of New South Wales, ADFA Canberra ACT 2600, Australia*

*Corresponding author: jan.forsman@compchem.lu.se

(Dated: 2025-01-02)

S1 Other polymer architectures

Here we will briefly consider other modelling options for the polyampholytes, which we recall also might serve as a crude description of ion clusters. One might anticipate a rather compact arrangement of an

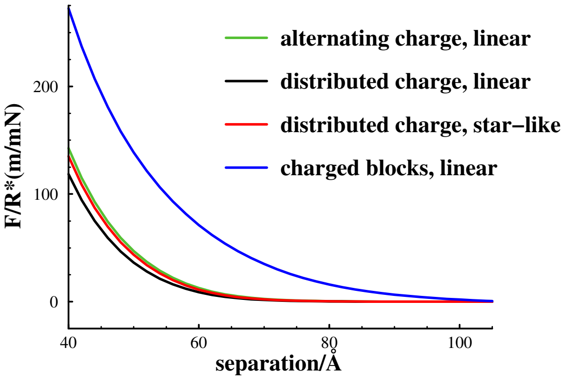

Figure 1: Interactions between charged surfaces, immersed in a solution containing 182mM 9-mer polyampholyte salts. The green curve are results using our reference model (main paper), i.e. composed of monomers with alternating charge. We also display (black) results using linearly connected chains with a single charge that is equally distributed between the monomers ($\pm e/9$ charge per monomer, resulting in monovalent chains). In the latter case, we furthermore show (red) corresponding interactions when the polymers have a 4-armed star structure, i.e. 4 branches connecting to a common central monomer. Finally, the blue curve shows interactions in the presence of polymers with a linear block charge structure (+ + + + + - - - - and + + + + - - - - -).

ion cluster, and a step in that direction would be to allow the polymers to become star-like, with several branches connecting to a common central monomer. However, we see in Figure 1 that the resulting interaction free energies are quite similar for branched and linear polymer architectures. Moreover, distributing the single net charge per chain uniformly among the monomers leads to quite similar results as for monovalent polympholytes in which the monomers have alternating charge. On the other hand, if the charges are collected into two separate blocks with opposing valency (one block containing one more monomer than the other), then the repulsive force become much stronger. One should then keep in mind that the construction of such polymers in a “real” solution might lead to folded structures, at least for

large degrees of polymerisation. The tendency to fold can be counteracted by the addition of simple salt, and interactions in the presence of such salt mixtures will be the focus of a future work.

Let us finally illustrate the relevance of excluded volume interactions, also when the monovalent charge is evenly distributed among the monomers, and when the polymers have a star-like structure. This is

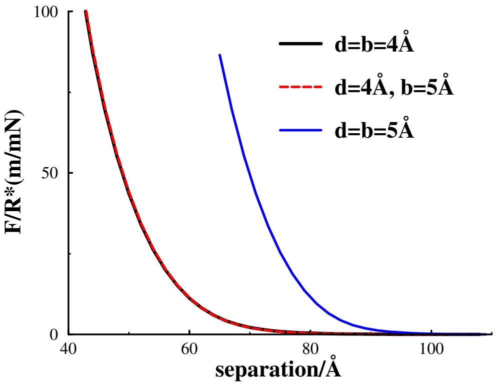

Figure 2: Interactions between charged surfaces, immersed in a solution containing 182mM 9-mer polyampholyte salts. Here, we consider chains with a 4-armed star structure, and various choices for the bond length (b) and monomer hard-sphere diameter (d).

illustrated in Figure 2, under conditions similar to those in Figure 1, i.e. 182mM monovalent 9-mer salt. We note that for a given monomer diameter, the bond length has a weak influence. On the other hand, increasing the hard-sphere monomer diameter generates a dramatically stronger repulsion.
